# Evaluation of the economic characteristics of the fruit of 45 superior *Camellia weiningensis* Y.K. Li. trees

**DOI:** 10.1371/journal.pone.0268802

**Published:** 2022-05-26

**Authors:** Lu Yang, Chao Gao, Hongli Wei, Li Long, Jie Qiu

**Affiliations:** Key laboratory of forest cultivation in plateau mountain of Guizhou province, Institute for Forest Resources and Environment of Guizhou, College of Forestry, Guizhou University, Guiyang, China; KGUT: Graduate University of Advanced Technology, ISLAMIC REPUBLIC OF IRAN

## Abstract

Reports related to *Camellia weiningensis* Y.K. Li. are rare. We evaluated the economic characteristics of the mature fruit of 45 superior *C*. *weiningensis* trees using principal component analysis (PCA) and gray correlation analysis, and identified excellent germplasms according to performance. PCA was employed to reduce the dimensions. PCA was performed for the original 15 indices of fruit diameter, fruit length, fruit shape, single-fruit weight, pericarp thickness, oil yield, fresh seed rate, dry seed rate, dry kernel rate and palmitic acid, stearic acid, linolenic acid, oleic acid, linoleic acid and arachidonic acid contents. According to the requirements of a cumulative contribution rate ≥ 80% and an eigenvector value > 1, six principle components were selected. These indices underwent weighted summation to establish a function model for comprehensive evaluation. Finally, the comprehensive rankings of the cultivars according to PCA were compared with those according to gray correlation analysis. The genetic variation coefficients of the 15 parameters ranged from 2.24% (oleic acid content) to 22.70% (single-fruit weight, with a range of 21.34 g). The top ten excellent cultivars with the highest comprehensive scores according to PCA and those according to gray correlation analysis were compared. According to PCA, oleic acid content, fruit diameter, fruit length, pericarp thickness, arachidonic acid content and dry seed rate can serve as representative evaluation indicators of *C*. *weiningensis*. The outcomes obtained based on PCA were basically consistent with those obtained based on gray correlation analysis. Finally, nine excellent cultivars were finally determined, i.e., WY-1, WY-6, WY-8, WY-25, WY-27, WY-30, WY-33, WY-35, WY-38 and WY-44. The results obtained in terms of crown yield were basically consistent with the outcomes of the comprehensive assessments, which indicates the reliability of the assessment methods used in this study.

## Introduction

*Camellia weiningensis* Y.K. Li., belonging to the *Camellia* L. genus of the Theaceae family, is a type of evergreen shrub or small tree. The species is endemic to regions of 1800–2700 m altitude in Weining County, Guizhou, China. The flowering time of *C*. *weiningensis* lasts from December to March of the following year, with the blossoming stage occurring in February. Its flowers are large with varying colors, and its fruit ripens between August and September. *Camellia weiningensis* has high economic value and is a rare economic tree in the high-altitude mountainous areas of northwestern Guizhou. In contrast to the widely distributed *C*. *oleifera* Abel, *C*. *weiningensis* has a high-altitude distribution and is characterized by resistance against cold, drought and barren soil; early maturation; a thin pericarp; a thin seed shell and high kernel and oil yields. Similar to common *Camellia* oil, *C*. *weiningensis* oil has a fatty acid content close to that of olive oil [1–3], and long-term consumption can prevent cardiovascular diseases [4, 5] and delay aging [6, 7]. In addition, the unique features of a low iodine content and high oleic acid content endow *C*. *weiningensis* oil with higher stability than oil from *Camellia* species growing in low-altitude regions [8, 9]. However, preferred *C*. *weiningensis* cultivars have long been lacking. The screening of existing resources can facilitate the identification of multiple excellent cultivars [10–13].

Methods that are currently often used for the comprehensive evaluation of economic characteristics include principal component analysis (PCA), gray correlation analysis, the rationalization-satisfaction method, and the technique for order preference by similarity to ideal solution (TOPSIS). All these methods have advantages as well as disadvantages. PCA has the advantage of not requiring artificial weighing, which makes the evaluation process objective. In PCA, redundant information can be removed through projection and dimension reduction, by which multiple observation indices are reduced to a small number of independent new indices to simplify the data structure. PCA is a multivariable statistical method for the comprehensive evaluation of multiple indices. Gray correlation analysis is a means used to determine the correlation degree between factors within the system based on their developmental similarities or differences, whose result can reflect the comprehensive performance of research subjects. Therefore, for multi-index, multi-factor analysis, the combined use of PCA and gray correlation analysis can comprehensively analyze the overall effect of each index and thus makes the screening results more scientific. To date, comprehensive evaluations of the economic characteristics of walnuts [14–18], wheat [19], rape [20] and rice [21] have been reported. Various methods have also been adopted to comprehensively evaluate the economic characteristics of common *Camellia* species. Chen [22] utilized PCA and the rationalization-satisfaction method combined with multidimensional value theory to analyze the nutritional components of oil from 11 *Camellia* cultivars and obtained their comprehensive evaluation-based order; furthermore, they found that the results from the two evaluation methods were largely consistent. Xie [23] comprehensively assessed 38 superior *Camellia* clones using dynamic TOPSIS (DTOPSIS), based on which they screened three excellent cultivars, i.e., “Huajin”, “Huaxin” and “Huashuo”. Jiang et al. [24] conducted screening among 28 single *Camellia* plants growing in the Dabie Mountain area in east Hubei using the fuzzy comprehensive evaluation method and identified three target superior trees for cultivar breeding. Wang et al. [25] used the rationalization-satisfaction method to evaluate 50 superior *C*. *oleifera* Abel plants as well as controls and screened 10 superior plants with high degrees of satisfaction. Nie et al. [26] adopted gray correlation analysis to comprehensively evaluate the oil from *C*. *oleifera* growing in different regions of Jiangxi and found that the qualities of the edible seed oil of plants growing in Yongfeng; Xinyu; Zhangshu; Yingtan Longhu Mountain; and Yuanzhou were the best. Zeng and Wang [27] conducted cultivar screening according to leaf anatomic structure based on the variation coefficient, correlation analysis and clustering analysis and comprehensively evaluated the drought resistance of the superior ‘Cenruan’ series clones of *C*. *oleifera* and ranked 9 superior clones according to drought resistance.

Due to its limited geographical distribution, *C*. *weiningensis* has not been the focus of systematic research since its discovery in 1979. Reports related to this species are rare, and its superior cultivars have not yet been selected. In recent years, our research group has conducted systematic research on *C*. *weiningensis* resources and has collected and stored more than 200 excellent germplasms. After years of observations and comparisons, 45 superior single plants with stable yield and satisfactory disease resistance were selected as potential superior single plants. In this study, we comprehensively evaluated the economic characteristics of the mature fruit of 45 single plants based on PCA and gray correlation analysis. We ranked these plants according to their economic performance and then selected the plants with satisfactory performance as excellent germplasms. The results of this study provide a foundation for the selection of excellent cultivars and crossbreeding of *C*. *weiningensis* to achieve the ultimate goal of creating new germplasms. Additionally, the results expand the available biological data on *C*. *weiningensis*.

## Materials and methods

### Experimental site and materials

All 45 superior *C*. *weiningensis* plants in this study are maintained at the *C*. *oleifera* Research Base of Guizhou University, Weining County, Bijie, Guizhou, China (long. 104°7′53′’E, lat. 27°11′53′’N). The average altitude of the base is 2,200 m. In this region, the daily temperature difference is large, the average annual rainfall is 909 mm, the average frost-free period is 180 d, the average annual sunshine hours is 1,812 h, and the average annual temperature is 11.5°C. The soil at the experimental site is slightly acidic, which is suitable for the growth of *C*. *weiningensis*.

**Fig 1:**
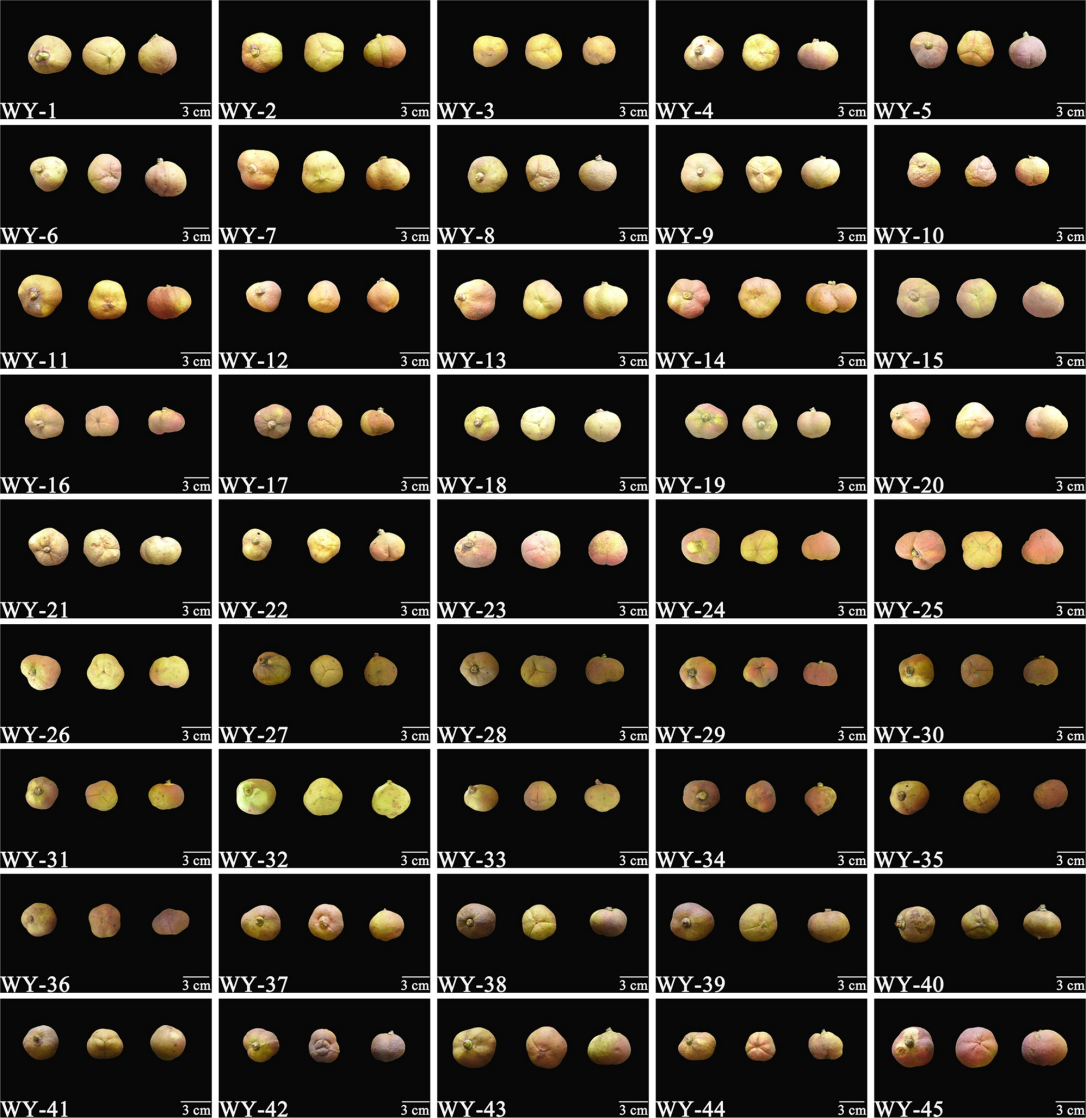
Fruit of 45 superior *C*. *weiningensis* plants (polar and equatorial views). Photos of three randomly selected mature fruits from the same tree. Two show the polar view, and one shows the equatorial view. The lower left corner of each panel provides the plant ID no., and the lower right corner indicates the scale (3 cm).

The criteria for the selection of the 45 superior *C*. *weiningensis* plants included healthy growth, a high seed-setting rate after natural pollination, a stable yield, and no diseases or insect pests. The plants that grew stably within three consecutive years were initially selected, and their height ranged from 1.5 m to 3.3 m, with a crown width of 140×160–265×270 cm. The plants were randomly designated as “WY-[serial number]”. In the fruit-ripening season (August 2020), all mature fruits were collected to measure the yield per plant. Among the mature fruits, 30 were randomly selected (identified based on slight cracking of the pericarp) were bagged and labeled (Fig 1). With 24 h, measurement of the phenotypic data and fresh seed weight were completed. After measurement, the fruits were subjected to sun drying. After all samples are collected, subsequent treatment, such as drying, was performed.

### Study design

After fruit collection, the fruit phenotypic and oil quality characteristics were determined and descriptive statistics and characteristic variation analysis were performed. Comprehensive assessments of the 15 economic characteristics of the mature fruit from each of the *C*. *weiningensis* plant were performed using PCA and gray correction analysis. The plants that ranked among the top ten according to both analyses were finally identified for the purpose of popularization.

## Determination of economic characteristics

Six sample fruits were randomly selected from each plant. For transverse diameter, two sides of each fruit were measured, and for pericarp thickness, measurement was performed at four points of each fruit. For the remaining characteristics, measurement was performed once. The experiment was repeated six times, and the averages were obtained for each characteristic.

(1) Longitudinal and transverse diameters. The longitudinal and transverse diameters and pericarp thickness of the fruit were measured with an electronic digital caliper (Mitutoyo 500-197-20; Japan; precision, 0.01 mm). The fruit shape index was calculated as follows:

Fruit shape index = transverse diameter/longitudinal diameter of the fruit.

(2) Single-fruit weight. The fruit weight was determined with an electronic balance (CP522C; precision, 0.01 g).

(3) Fresh seed rate. Fresh seeds were removed from the weighed fresh fruit and then weighed with an electronic balance. The fresh seed rate was calculated as follows:

Fresh seed rate = fresh weight of the seeds/ fresh weight of the fruit×100%

(4) Dry seed rate. Fresh seeds were dried to a constant weight and then weighed with an electronic balance, and the calculation equation was as follows:

Dry seed rate = weight of the dry seeds/weight of the fresh seeds×100%

(5) Dry kernel rate. Kernels were removed from the dried seeds, dried to constant weight, and then weighed with an electronic balance. The calculation equation was as follows:

Dry kernel rate = weight of the dry kernels/weight of the dry seeds×100%.

(6) Crown yield. The crown size of each plant was measured, and then the yield of the plant was divided by the size to obtain the crown yield (kg/m^2^).

### Oil extraction

Oil was extracted using the Soxhlet extractor method [28]. Specifically, kernels were dried at 40°C for 24 h and then ground. Ten grams (raw weight) of each sample was wrapped in dried 12 cm filter paper (weight, W_0_) and then dried to constant weight. After cooling, the filter paper and the sample were accurately weighed to obtain the total weight W_1_. The filter paper package with the sample was placed into a Soxhlet extractor for circular extraction for 10 h with petroleum ether. The package was then removed, dried to a constant weight and then accurately weighed. The total weight of the filter paper and residue W_2_ was obtained. The oil yield was calculated as follows:

$$Oil\;yield=(W_{1}‐W_{2})/(W_{1}‐W_{0})\times 100\%$$

### Fatty acid composition and content determination

The procedures were as follows:

1. An oil sample (100 mg, accurately weighed) was added to a conical flask.
2. Methanol (40 mL) and 2 mL of 0.5 mol/L potassium hydroxide-methanol solution were added to the oil sample. The sample underwent reflux at 75°C. (During reflux, the flask was constantly shaken until the oil disappeared and the solution became transparent.)
3. The conical flask was removed and then cooled in chilled water to room temperature.
4. The solution in the flask was transferred into a separating funnel. The flask was washed with 20 mL of n-heptane, and the washing liquid was transferred into the funnel. Then, 20 mL of chromatography-grade pure water was added to the funnel. The solution was mixed well and then allowed to stand for stratification. (The upper layer was the ester layer, and the lower layer was the aqueous layer.) The lower layer was extracted with 20 mL of n-heptane. The extracts were combined and then washed with pure water 2–3 times until the discarded water appeared neutral. The ester layer was isolated.
5. An appropriate volume of anhydrous sodium sulfate was added to the ester solution. After filtering, the drying agent was washed with a small volume of n-heptane. The washing liquid and ester solution were merged. Afterwards, the volume of the obtained solution was increased with n-heptane to 100 mL for later use. The conditions for gas chromatography were as follows: detection, flame ionization detection (FID); column type and specification, SP2340 and 60 m × 0.25 mID 111/11 ×0.2 μm; temperature program, 50°C (initial temperature) for 2 min followed by 170° C (10° C/min) for 10 min, 180°C (2°C/min) for 10 min and 220°C (4°C/min) for 22 min; inlet temperature, 250°C; and detector temperature, 300°C. Nitrogen was used as the carrier gas, with a separating ratio of 1:50 and a sample feed of 1 μL.

The components of fatty acids were determined based on comparisons with the retention times of samples of different fatty acid standards. The relative content of each component was calculated using the area normalization method. The experiment was repeated three times, and an average value of each component was obtained [25].

### Statistical analysis

Averages, maximum values, minimum values, means, standard deviations, variation coefficients, and the matrix of comprehensive scores were calculated with Excel 2019. SPSS20.0 was used for PCA, Origin for correlation analysis and Photoshop 2019 for image processing. Gray correlation analysis was performed to comprehensively evaluate the fruit characters of different excellent plants according to the observed values of typical indices, and the gray correlation coefficient was calculated as follows:

$$\xi_{i}(k)=\frac{\underset{i}{\min}\;\underset{k}{\min}|X_{0}(k)-X_{i}(k)|+\rho *\underset{i}{\max}\;\underset{k}{\max}\left|X_{0}(k)-X_{i}(k)\right|}{|X_{0}(k)-X_{i}(k)|+\rho *\underset{i}{\max}\;\underset{k}{\max}\left|X_{0}(k)-X_{i}(k)\right|}$$

where *ρ* represents the resolution coefficient (set at 0.5 here). The correlation coefficient of each index of the plants was obtained. For each plant, the correlation coefficients of the characters were summed up, and an average was obtained, which was used as the gray correlation degree (*r*_*i*_) of the plant. All plants were ranked according to their gray correlation degrees, and a higher *r*_*i*_ value indicated more excellent fruit characteristics.

## Results

### Variation analysis of the fruit and seed characteristics

The fruit characters of the 45 superior plants are summarized in Table 1. The genetic variation coefficients of the 15 characteristics of the plants ranged from 2.24% to 22.70%. The genetic variation coefficients of fruit diameter, fruit height, fruit shape index, fresh seed rate, dry seed rate, dry kernel rate, palmitic acid, oleic acid and arachidonic acid were small, which all fell between 0% and 15%, while single fruit weight, pericarp thickness, oil content, linoleic acid and linolenic acid had great genetic variations (all between 15% and 30%). Single fruit weight showed the greatest variation, with a range of 21.34 g and a genetic variation coefficient reaching up to 22.70%. The genetic variation coefficient of oil content was also large, which was 13.11%. These results indicated that both single fruit weight and oil content had abundant hereditary characteristics. Linoleic acid, stearic acid and pericarp thickness followed single fruit weight and oil content, whose variation coefficients were 8.40%, 17.75% and 17.36%, respectively. In contrast, the variation coefficients of oleic acid, fruit diameter and arachidonic acid were low, which were 2.24%, 6.91% and 9.10%, indicating that these characters were largely stable.

**Table 1:** Seed and fruit characteristics of the fruit of 45 superior *Camellia weiningensis* trees.

Index	Min.	Max.	Range	Average	Kurtosis	Skewness	S.D.	CV (%)
Fruit diameter (mm)	28.22	38.98	10.76	33.23	0.14	0.18	2.30	6.91
Fruit length (mm)	20.22	40.92	20.7	27.63	5.58	1.22	3.24	11.73
Single fruit weight (g)	10.4	31.74	21.34	16.68	5.05	1.65	3.79	22.70
Pericarp thickness (mm)	2.04	4.27	2.23	2.87	0.11	0.62	0.50	17.36
Fruit shape	0.69	1.73	1.04	1.22	2.98	0.16	0.16	13.49
Oil content (%)	29.55	63.84	34.29	44.41	-0.98	-0.11	8.70	19.60
Fresh seed rate (%)	27.18	58.97	31.79	43.75	0.46	-0.56	6.43	14.69
Dry seed rate (%)	40.69	72.92	32.23	61.79	2.60	-1.38	6.81	11.02
Dry kernel rate (%)	23.49	57.55	34.06	46.27	2.11	0.35	6.64	14.36
Palmitic acid (%)	8.74	12.40	3.66	10.30	-0.51	0.47	0.92	8.90
Stearic acid (%)	1.99	4.59	2.60	3.04	1.40	0.83	0.54	17.75
Oleic acid (%)	74.12	81.45	7.33	78.39	-0.02	-0.34	1.76	2.24
Linoleic acid (%)	4.68	10.43	5.75	7.22	-0.12	0.25	1.33	18.40
Linolenic acid (%)	0.45	0.83	0.38	0.61	0.28	0.77	0.10	15.87
Arachidonic acid (%)	0.36	0.55	0.19	0.45	0.44	0.54	0.04	9.10

Notes: Min., minimum; Max., maximum; S.D., standard deviation; CV, coefficient of genetic variation

From the perspective of skewness coefficient, fruit diameter, fruit length, single fruit weight, pericarp thickness, fruit shape, dry kernel yield, palmitic acid, stearic acid, linoleic acid, linolenic acid and arachidonic acid all had positive values, which indicated that their distribution curves deviated to the left (small diameter; the deviation degrees were ranked from top to bottom as follows: Single fruit weight > longitudinal diameter > stearic acid > linolenic acid > peel thickness > arachidonic acid > palmitic acid > dry kernel yield > linoleic acid > fruit diameter > fruit shape). In terms of kurtosis coefficient, the coefficients of all these characters but palmitic acid and linoleic acid were positive. This finding indicated that these characters had concentrated distribution, whose distribution curves were steeper than a normal distribution, with a sharp peak appearance. Fruit length had the greatest kurtosis coefficient, whose distribution curve were therefore the steepest. The kurtosis coefficients of palmitic acid and linoleic acid were negative, with a relatively discrete character distribution. The skewness coefficients of oil content, fresh seed yield, dry seed yield and oleic acid were also negative. These results indicated that the distribution curves of these characters were right-biased. The kurtosis coefficients of fresh seed rate and dry seed rate were positive, whose distributions were relatively concentrated.

### Correlations among characteristics

**Fig 2:**
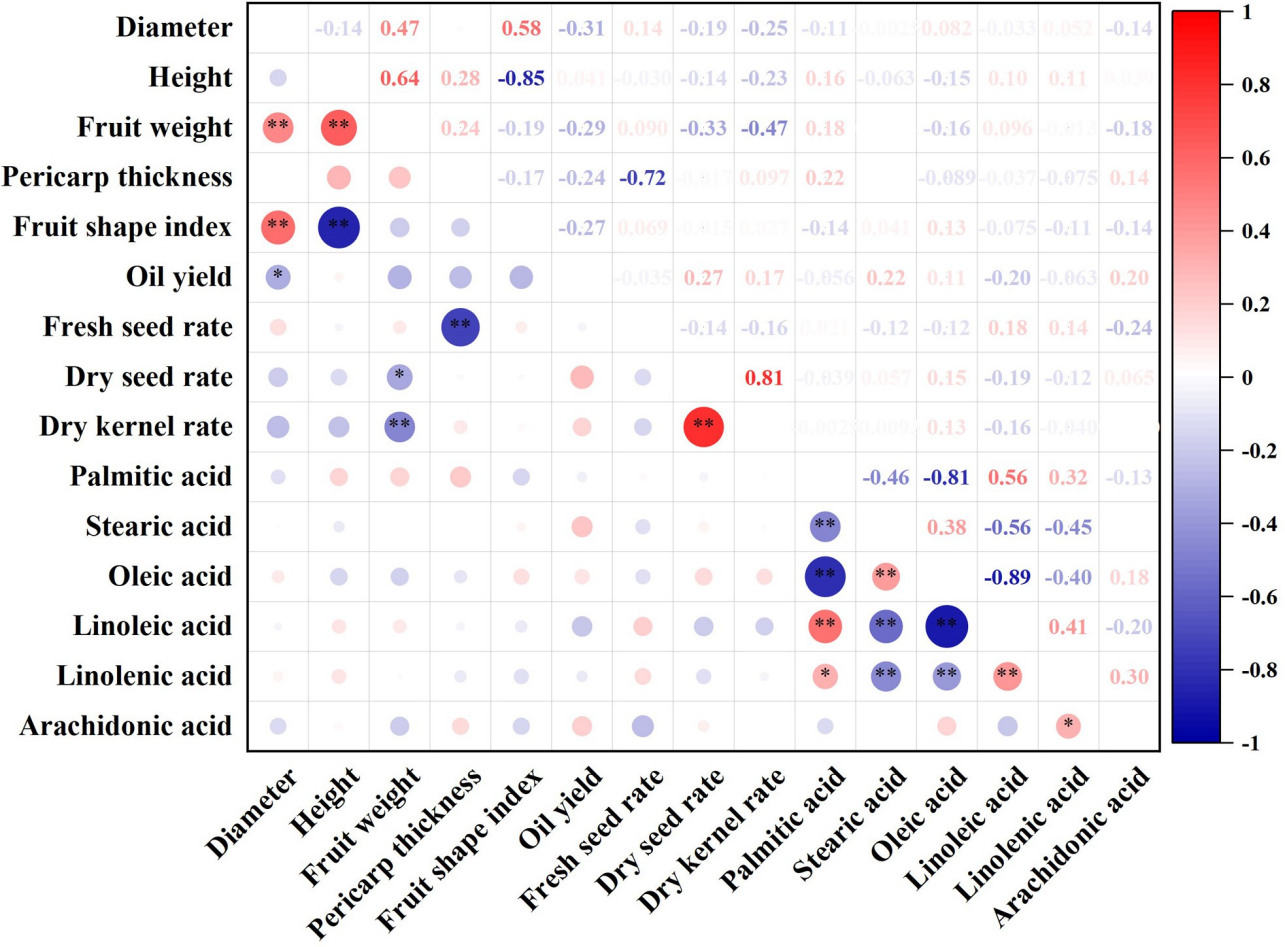
Correlation analysis of the characters of *C*. *weiningensis* using origin. Notes: *significantly correlated (P<0.05); **very significantly correlated (P<0.01).

The results of the correlation analysis of the 15 characteristics were shown in Fig 2. Both diameter and height had very significant positive correlations with fruit weight. Fruit weight had a very significant negative correlation with dry kernel rate and a significant negative correlation with dry seed rate, and pericarp thickness had a very significant negative correlation, which indicated that a heavier fruit with a thicker pericarp had a lower seed rate.

Palmitic acid content exhibited very significant negative correlations with the contents of stearic acid and oleic acid. The content of this fatty acid also had a very significant positive correlation with linoleic acid content and a significant positive correlation with linolenic acid content. Stearic acid content had a very significant positive correlation with oleic acid content and very significant negative correlations with linoleic acid and linolenic acid contents. Oleic acid content showed very significant negative correlations with linoleic acid and linolenic acid contents, and linoleic acid and linolenic acid contents showed a very significant positive correlation with each other. The remarkable correlations among the components of fatty acids suggested that the content of a certain component could be evaluated based on that of another component. In addition, the degrees of correlation among the characteristics were different, which indicated information overlap among the investigated characteristics; therefore, PCA was required to reduce the number of variables.

### PCA

PCA was performed on the 15 fruit character indices of the 45 *C*. *weiningensis* plants (Table 2). Based on the criteria of cumulative contribution rate (CCR) ≥80% and eigenvalue (E) >1, six principal components were extracted. The CCR of the six components reached 83.06%, which meant that these components contained most of the information of the original 15 indices. These six principal components were then used as the comprehensive assessment indices for the purpose of dimension reduction.

**Table 2:** Eigenvector, eigenvalues and variance contribution rates of the principal components.

	Principal component					
	1	2	3	4	5	6
Fruit diameter (mm)	0.074	-0.738	0.059	0.290	0.055	0.429
Fruit length (mm)	0.435	0.343	0.719	-0.255	-0.138	0.272
Single fruit weight (g)	0.471	-0.328	0.645	0.038	-0.215	0.336
Pericarp thickness (mm)	0.124	0.310	0.478	0.757	0.022	-0.075
Fruit shape	-0.268	-0.669	-0.506	0.405	0.081	-0.043
Oil content (%)	-0.312	0.452	-0.047	-0.466	0.016	-0.113
Fresh seed rate (%)	0.200	-0.398	-0.325	-0.682	-0.16	0.252
Dry seed rate (%)	-0.425	0.513	-0.345	0.151	-0.314	0.457
Dry kernel rate (%)	-0.402	0.531	-0.436	0.264	-0.279	0.362
Palmitic acid (%)	0.731	0.282	-0.225	0.199	-0.171	-0.078
Stearic acid (%)	-0.591	-0.114	0.374	-0.099	-0.183	-0.228
Oleic acid (%)	-0.843	-0.175	0.291	-0.083	0.144	0.198
Linoleic acid (%)	0.820	0.061	-0.365	0.020	-0.064	-0.142
Linolenic acid (%)	0.511	0.155	-0.267	-0.083	0.571	0.331
Arachidonic acid (%)	-0.167	0.338	0.125	0.004	0.804	0.120
Eigenvalue	3.538	2.488	2.333	1.727	1.337	1.037
VCR (%)	23.586	16.584	15.551	11.513	8.915	6.912
CCR (%)	23.586	40.171	55.721	67.235	76.150	83.062

Notes: VCR, variance contribution rate; CCR, cumulative contribution rate.

The loading matrix of principal components can reflect the contribution of each characteristic to the principal components; moreover, it can reflect the main information conveyed by each principal component. As shown in Table 2, the first principal component contained the information about palmitic acid, stearic acid, oleic acid and linoleic acid, with a variance contribution rate of 23.586%. Oleic acid showed the most significant correlation with the first principal component, whose factor load is -0.843. The second principal component contained the information about fruit diameter, fruit shape and dry kernel rate, with a variance contribution rate of 16.584%. Fruit diameter showed the most significant correlation with the second principal component, with a factor load of -0.738. The third principal component contained the information about fruit length and single fruit weight, with a variance contribution rate of 15.551%. Fruit length showed more significant correlation with the third principal component, with a factor load of 0.719. The fourth principal component contained the information about pericarp thickness, oil content and fresh seed rate, with a variance contribution rate of 11.513%. Pericarp thickness showed the most significant correlation with the fourth principal component, with a factor load of 0.757. The fifth principal component contained the information about linolenic acid and arachidonic acid, with a variance contribution rate of 8.915%. Arachidonic acid showed a more significant correlation with the fifth principal component, with a factor load of 0.804. The sixth principal component contained the information of dry seed rate, whose factor load was 0.457. Finally, the six indices, i.e., oleic acid, fruit diameter, fruit length, pericarp thickness, arachidonic acid and dry seed rate, were finally taken as the representative indices to comprehensively assess the *C*. *weiningensis* trees.

### Comprehensive evaluation based on PCA and gray correlation analysis

Data of the 45 plants were standardized, and their scores on the six principal components were obtained (Table 3). These principal components reflected the comprehensive conditions of *C*. *weiningensis* fruit at different levels, which could not be fulfilled by any single principal component. Based on the PCA scores, with the variance contribution rate corresponded by each principal component as the weight, a comprehensive evaluation function was constructed, and the function model *Z* was as follows:

$$Z=0.284Y_{1}+0.200Y_{2}+0.187Y_{3}+0.139\;Y_{4}+0.107\;Y_{5}+0.083\;Y_{6}$$

**Table 3:** Scores of the 45 *C*. *weiningensis* trees on the six principal components.

Tree no.	PC1	PC2	PC3	PC4	PC5	PC6
WY-1	0.352	2.133	0.700	2.176	0.235	0.010
WY-2	0.444	-1.273	0.536	0.652	0.711	-0.101
WY-3	-1.086	0.804	0.547	-0.772	-0.52	-1.263
WY-4	-0.197	-1.112	-1.11	1.378	0.084	-0.295
WY-5	0.303	-0.733	0.946	0.330	1.895	-1.161
WY-6	-0.209	0.733	1.139	1.144	-0.418	-0.120
WY-7	-1.413	-0.163	0.568	0.508	-0.098	-1.340
WY-8	2.028	1.620	-0.895	0.539	2.343	-0.488
WY-9	0.522	-0.452	-1.531	0.032	-0.048	-0.653
WY-10	0.379	0.692	0.054	0.699	-0.229	-1.141
WY-11	-0.948	-0.925	-0.734	-0.216	-0.238	0.526
WY-12	0.105	0.685	-1.263	-1.118	-0.751	-0.307
WY-13	-0.852	-0.673	0.617	0.485	0.104	1.107
WY-14	-1.101	-0.045	0.628	-0.064	-0.608	1.261
WY-15	-0.440	-1.361	-1.554	1.885	0.212	0.191
WY-16	-0.829	-1.031	0.124	0.530	-1.567	-0.590
WY-17	-0.445	0.438	0.69	0.959	-0.974	0.078
WY-18	0.800	-1.697	0.123	0.594	0.067	-0.909
WY-19	1.156	0.050	-0.25	-0.577	-0.589	1.170
WY-20	-2.255	0.86	-0.185	1.334	-0.032	-0.185
WY-21	-1.948	1.152	0.414	-0.962	0.952	-0.742
WY-22	-0.913	-0.289	0.664	-1.058	-0.940	0.37
WY-23	0.678	-1.57	-0.051	-0.171	0.476	-0.301
WY-24	1.215	-1.724	0.527	0.112	-0.332	1.753
WY-25	-0.984	1.33	1.187	0.806	0.021	1.349
WY-26	-0.431	-1.834	1.462	-2.088	1.451	-1.948
WY-27	1.667	1.019	-0.62	0.066	1.391	0.094
WY-28	1.139	0.348	-1.363	0.851	-1.099	-1.832
WY-29	0.815	-1.777	-0.063	0.748	-0.550	0.809
WY-30	0.469	-0.101	1.254	1.275	0.321	0.177
WY-31	0.836	0.146	-1.542	-0.151	-0.788	-2.065
WY-32	-0.095	1.034	-1.21	-0.887	-0.889	-0.294
WY-33	2.413	0.856	3.705	-0.741	-1.727	-0.766
WY-34	-1.874	0.603	-0.14	0.56	1.433	-0.335
WY-35	0.268	0.965	-0.572	-0.846	2.402	0.900
WY-36	-0.664	0.070	0.106	-1.459	0.78	0.078
WY-37	-0.533	-0.443	-0.703	-0.915	0.029	0.200
WY-38	0.945	1.050	-0.151	-0.143	-0.703	0.879
WY-39	0.151	0.785	-1.503	-0.83	-1.748	0.364
WY-40	-0.254	-0.892	0.044	0.452	-0.743	0.869
WY-41	-0.131	0.082	-0.933	-2.003	-0.312	1.594
WY-42	0.111	0.488	0.206	-1.558	-0.608	-0.057
WY-43	0.099	-0.211	0.091	-1.401	0.081	-0.698
WY-44	0.477	-0.336	0.349	-0.548	1.925	1.940
WY-45	0.231	0.696	-0.307	0.393	-0.400	1.872

The comprehensive scores (*F*) of the economic characteristics of the fruit of 45 superior *C*. *weiningensis* trees were finally obtained, according to which the trees were ranked and superior plants were screened.

In addition, based on the 15 indices of *C*. *weiningensis*, the correlation coefficients of each index were calculated. The correlation coefficients of the indices were accumulated and an average was obtained. The trees were ranked according to the averages they obtained for comprehensive evaluation.

The rankings of the comprehensive quality of the fruit of the 45 *C*. *weiningensis* plants based on PCA and gray correlation analysis are summarized in Table 4. According to the PCA, the top ten plants were ordered as follows: WY-33>WY-8>WY-1>WY-27>WY-30>WY-25>WY-38>WY-44>WY-6>WY-35. According to the gray correlation analysis, the order of the top ten plants was WY-1>WY-8>WY-38>WY-25>WY-30>WY-33>WY-6>WY-27>WY-35> WY-19. The rankings based on the two analytical methods were subjected to correlation analysis, and the correlation coefficient was 0.535, showing a very significantly positive correlation (P<0.01). Therefore, the evaluation results based on these two methods had satisfactory consistency and thus verified each other. Although the same plant might exhibit a difference in the rankings according to different evaluation methods, this difference was caused by the different weights assigned to each index during analyses. Additionally, the overall rankings of the plants based on the two methods were comparable, which indicated overall consistent evaluation trends according to the two methods. Based on these results, nine *C*. *weiningensis* plants whose fruit characters ranked in the top ten, according to both evaluation methods, were selected for promotion, which included WY-1, WY-6 WY-8, WY-25, WY-27, WY-30, WY-33, WY-35, WY-38 and WY-44. The detailed economic characteristics of the nine *C*. *weiningensis* plants are summarized in Table 5.

**Table 4:** Comprehensive evaluation scores of the 45 *C*. *weiningensis* plants and their rankings based on the two analytical methods.

Plant no.	PCA		Gray correlation analysis	
	*F* (comprehensive score)	Ranking	Average	Ranking
WY-1	0.986	3	1	1
WY-2	0.13	16	0.789	23
WY-3	-0.313	33	0.794	19
WY-4	-0.31	32	0.806	14
WY-5	0.269	12	0.795	17
WY-6	0.405	9	0.825	7
WY-7	-0.379	39	0.774	34
WY-8	1.018	2	0.84	2
WY-9	-0.283	29	0.787	25
WY-10	0.234	14	0.745	43
WY-11	-0.603	45	0.78	28
WY-12	-0.331	34	0.814	12
WY-13	-0.091	21	0.788	24
WY-14	-0.174	24	0.774	33
WY-15	-0.387	40	0.802	15
WY-16	-0.561	44	0.728	44
WY-17	0.126	17	0.785	27
WY-18	-0.075	20	0.766	38
WY-19	0.245	13	0.818	10
WY-20	-0.336	35	0.779	29
WY-21	-0.339	36	0.761	40
WY-22	-0.41	41	0.754	41
WY-23	-0.129	23	0.775	31
WY-24	0.224	15	0.761	39
WY-25	0.435	6	0.835	4
WY-26	-0.512	43	0.716	45
WY-27	0.727	4	0.822	8
WY-28	-0.013	18	0.795	18
WY-29	-0.023	19	0.772	35
WY-30	0.574	5	0.833	5
WY-31	-0.298	31	0.766	37
WY-32	-0.289	30	0.817	11
WY-33	1.198	1	0.827	6
WY-34	-0.234	26	0.775	32
WY-35	0.376	10	0.822	9
WY-36	-0.268	28	0.787	26
WY-37	-0.479	42	0.768	36
WY-38	0.428	7	0.835	3
WY-39	-0.353	37	0.799	16
WY-40	-0.187	25	0.807	13
WY-41	-0.375	38	0.794	20
WY-42	-0.119	22	0.789	22
WY-43	-0.241	27	0.777	30
WY-44	0.424	8	0.751	42
WY-45	0.315	11	0.791	21

**Table 5:** Detailed economic characteristics of the fruit of the nine superior *C*. *weiningensis* plants.

Plant no.	Fruit diameter (mm)	Fruit length (mm)	Single fruit weight (g)	Pericarp thickness (mm)	Fruit shape	Fresh seed rate (%)	Dry seed rate (%)	Dry kernel rate (%)
1	31.02±0.90e	31.09±1.06bc	14.16±0.71f	4.27±0.05a	1.00±0.06g	29.92±0.10f	65.99±1.76bc	55.50±1.18a
6	35.02±0.44ab	29.59±0.9bcde	19.85±0.90c	3.65±0.05c	1.18±0.02bc	35.46±0.87e	67.08±1.07b	49.40±0.45c
8	31.23±0.95e	27.66±1.01e	14.8±0.01ef	3.46±0.04e	1.13±0.01cd	41.58±1.38c	59.60±2.14d	44.10±0.89de
25	33.89±1.61bcd	31.51±1.30b	18.08±0.10d	3.49±0.04de	1.08±0.01ef	30.85±0.99f	72.58±1.99a	53.29±0.91ab
27	32.49±1.24de	29.39±1.03cde	15.64±1.29ef	2.80±0.05g	1.11±0.00de	38.52±0.93d	58.71±1.48d	43.88±1.20de
30	34.63±1.18abc	29.76±1.11bcd	20.10±0.24bc	3.85±0.10b	1.16±0.00bc	37.74±1.54d	59.27±1.27d	42.34±1.34e
33	28.22±0.63f	40.92±1.80a	31.74±0.75a	3.61±0.15cd	0.69±0.01h	41.35±0.80c	50.30±0.75e	32.60±1.02f
35	31.66±1.07e	28.16±1.00de	14.02±0.47f	2.49±0.11h	1.12±0.00cd	44.70±1.12b	63.71±0.93c	45.73±0.88d
38	32.92±0.92cde	31.29±0.71bc	17.49±0.11d	3.13±0.01f	1.05±0.01f	44.49±0.60b	65.08±1.08bc	51.32±1.11bc
44	36.09±1.09a	29.3±0.65cde	21.10±0.10bc	2.74±0.02g	1.23±0.01a	48.99±0.99a	60.69±1.49d	42.92±2.92e

Plant no.	Oil content (%)	Palmitic acid (%)	Stearic acid (%)	Oleic acid (%)	Linoleic acid (%)	Linolenic acid (%)	Arachidonic acid (%)
1	36.30±0.60d	11.16±0.16c	2.33±0.01cd	78.01±0.42bcd	7.45±0.06d	0.57±0.01d	0.49±0.01bc
6	50.67±1.22b	10.88±0.33cd	4.52±0.17a	76.43±0.20cd	7.11±0.22e	0.56±0.02de	0.49±0.02bc
8	48.00±0.80c	12.06±0.20a	2.14±0.02de	74.12±0.84e	10.30±0.10a	0.83±0.01a	0.55±0.03a
25	54.75±1.50a	9.22±0.20f	3.10±0.03b	80.42±1.19a	6.20±0.08g	0.59±0.01d	0.47±0.02cd
27	47.31±0.99c	11.93±0.41ab	1.99±0.02e	76.07±1.07d	8.72±0.07b	0.83±0.03a	0.47±0.01cd
30	35.67±1.05de	10.08±0.08e	3.13±0.03b	78.31±1.06bcd	7.40±0.17d	0.64±0.02c	0.45±0.03d
33	34.22±1.12e	11.42±0.42bc	3.08±0.03b	76.33±1.31d	8.24±0.04c	0.52±0.02e	0.41±0.02e
35	48.78±1.19c	10.14±0.02e	2.31±0.03cd	78.65±1.10b	7.56±0.06d	0.80±0.02a	0.54±0.02a
38	51.67±0.78b	11.03±0.44c	2.36±0.02c	77.20±1.00bcd	8.31±0.03c	0.69±0.03b	0.41±0.01e
44	47.25±0.67c	10.38±0.38de	3.10±0.30b	78.46±1.46b	6.71±0.19f	0.83±0.05a	0.52±0.02ab

Notes: Data are presented as the mean± standard deviation; A different letter in the same column indicates a very significant difference (P<0.01) in the same column according to analysis of variance.

The obtained outcomes based on the PCA and gray correlation analysis were compared with the actually measured data. WY-33, which ranked first according to the PCA, ranked the first both in fruit length and single fruit weight among the plants, and WY-1, which occupied the first position according to the gray correlation analysis, ranked the first in pericarp thickness and its dry kernel rate nearly reached the highest value among all investigated plants. WN-8, which ranked the second according to both methods, ranked the first in palmitic acid, linoleic acid and arachidonic acid. The nine germplasm plants selected based on both methods exhibited excellent performance in terms of all characters. Therefore, the outcomes obtained based on PCA and gray correlation analysis were basically consistent with the actually measured data, which indicated that these nine germplasms had the potential to be applied in actual production.

### Comparison with the five-year average crown yield

The obtained outcomes based on two analytical methods in this study were compared with five-year average crown yields. As shown in Table 6, WY-33, which ranked first according to the five-year crown yield, ranked first according to the PCA and the sixth according to the gray correlation analysis. WY-1, which ranked the second according to the five-year crown yield, ranked the third according to the PCA and the first according to the gray correlation analysis. There existed inconsistencies. For instance, WY-16 ranked the 35th in terms of the crown yield and the 44th according to both analytical methods. Although the overall fruit quality of this plant was relatively inferior, its performance in yield was not so poor. Also, although some plants had comparatively low yields, their overall fruit quality was relatively satisfactory (e.g., WY-28 and WY-29). Despite the inconsistencies, the assessment outcomes based on PCA and gray correlation analysis were consistent with the actual yields to a certain extent, which indicated the reliability of the assessment outcomes in this study. In addition, based on the results of the comparison with the five-year average crown yield, the PCA outperformed the gray correlation analysis.

**Table 6:** Comparison between the rankings according to principal component analysis and gray correlation analysis and those in terms of five-year average crown yield.

Plant no.	Crown yield (kg/m^2^)	Yield ranking	PCA ranking	GCA ranking
WY-1	4.35±0.93	2	3	1
WY-2	2.53±0.56	19	16	23
WY-3	1.66±0.33	33	33	19
WY-4	2.33±0.60	27	32	14
WY-5	2.76±0.23	15	12	17
WY-6	3.26±0.76	13	9	7
WY-7	1.37±0.21	43	39	34
WY-8	4.33±0.55	3	2	2
WY-9	1.33±0.28	44	29	25
WY-10	2.97±0.43	14	14	43
WY-11	1.38±0.28	42	45	28
WY-12	2.35±0.32	26	34	12
WY-13	2.46±0.33	23	21	24
WY-14	2.47±0.61	22	24	33
WY-15	1.42±0.31	41	40	15
WY-16	1.58±0.32	35	44	44
WY-17	2.21±0.56	29	17	27
WY-18	2.54±0.53	18	20	38
WY-19	3.40±0.17	10	13	10
WY-20	1.70±0.09	32	35	29
WY-21	1.65±0.23	34	36	40
WY-22	1.31±0.14	45	41	41
WY-23	2.52±0.23	21	23	31
WY-24	2.68±0.25	16	15	39
WY-25	4.13±0.78	5	6	4
WY-26	1.45±0.45	39	43	45
WY-27	3.95±0.34	6	4	8
WY-28	3.28±0.33	12	18	18
WY-29	2.55±0.18	17	19	35
WY-30	4.31±0.27	4	5	5
WY-31	2.21±0.31	30	31	37
WY-32	1.96±0.42	31	30	11
WY-33	4.71±0.50	1	1	6
WY-34	1.52±0.21	37	26	32
WY-35	3.53±0.12	8	10	9
WY-36	2.39±0.23	25	28	26
WY-37	1.47±0.26	38	42	36
WY-38	3.76±0.34	7	7	3
WY-39	1.53±0.09	36	37	16
WY-40	2.43±0.23	24	25	13
WY-41	1.43±0.25	40	38	20
WY-42	2.52±0.45	20	22	22
WY-43	2.32±0.23	28	27	30
WY-44	3.51±0.54	9	8	42
WY-45	3.32±0.78	11	11	21

Notes: Data on crown yield are presented as the mean ± standard deviation. PCA, principal component analysis; GCA, gray correlation analysis.

## Discussion

The fatty acids of edible vegetable oil are mainly composed of monounsaturated fatty acids (MUFAs) and polyunsaturated fatty acids (PUFAs). Compared with oils from other common oil crops in China, such as soybean (MUFA content, 24.63; PUFA content, 58.54), rapeseed oil (MUFA content, 65.03; PUFA content, 26.89), corn oil (MUFA content, 30.33; PUFA content, 54.37), sunflower seed oil (MUFA content, 26.92; PUFA content, 61.18), peanut oil (MUFA content, 42.34; PUFA content, 35.95) [29], *C*. *weiningensis* oil showed much higher MUFA and PUFA values (88.65 and 7.88, respectively). Higher intake of monounsaturated fatty acids than saturated fatty acids benefits blood sugar and blood lipid control, which can partially reduce the risks of hyperinsulinemia, pancreatitis and β-cell dysfunction induced by excessive intake of saturated fatty acids and the risk of invasion of renal cell cancer to renal cells [30]. Presently, cardiovascular and cerebrovascular diseases and diabetes have high incidence; therefore, *C*. *weiningensis* oil has great potential for promotion. Furthermore, with the improvement of people’s living standards, the demand for edible vegetable oil is increasing. China has become a large consumer and importing country of edible vegetable oil; approximately 70% of the oil consumed in the country is imported. At present, edible vegetable oil in China is mainly extracted from rapeseed, peanut, soybean and other herbal oil crops. The country is unlikely to develop woody oil production by sacrificing cultivated land. However, developing woody oil crops in mountainous areas may be a feasible solution to this issue. This solution may not only reduce the current reliance of China on import for edible oil but also conserve areas of cultivated land for grain crops, thereby maintaining food security in China [31].

Current methods adopted for the comprehensive assessment of fruit economic characteristics mainly include PCA, gray correlation analysis, DTOPSIS and the rationalization-satisfaction method. PCA and grey correlation degree are both commonly used multivariate statistical methods. Although their analysis principles are different, they can both be used to comprehensively and objectively evaluate the multiple characters of fruit. PCA offers advantages in that it does not entail artificial weighting of each characteristic and the contribution rates of the principal components function as the weights used in other evaluation methods; these features enable the entire assessment process to be objective. Gray correlation analysis can solve the difficulty in index selection when the indices are in an excessively large number, and thus realizes comprehensive, all-round evaluation. Based on the final comprehensive scores and ordering outcomes of the superior plants in this study, the superior *C*. *weiningensis* plants tended to be those with heavy single fruits, a high kernel rate and high palmitic acid, oleic acid, linoleic acid and arachidonic acid contents.

Oil yield and fatty acid composition are important indices for the comprehensive assessment of the economic characteristics of *C*. *oleifera*. The oil yield and fatty acid composition of *C*. *weiningensis* differ to various degrees from those of Hainan *C*. *oleifera* [32]: Both the average oil yield and average oleic acid content of *C*. *weiningensis* are higher than those of *C*. *oleifera*. Moreover, the variation coefficients of their characteristics also differ. The variation coefficients of *C*. *weiningensis* are generally lower than 0.1%, whereas in Hainan *C*. *oleifera*, those of linoleic acid (46.12%), oleic acid (6.39%), palmitic acid (13.41%), stearic acid (21.53%) and linolenic acid contents (39.7%) are remarkable. Growth regions and cultivar types have tremendous impacts on the properties of tea seed oil, such as oil components and fatty acid composition [33]. Such impacts are mainly caused by precipitation, illumination, temperature, altitude and topography [34–38]. Weining County has a high average altitude, and the oleic acid, linoleic acid and linolenic acid contents of *C*. *weiningensis* were 78.35, 7.26 and 0.61, respectively. The contents of these three fatty acids in *C*. *oleifera* have been measured in Liuyang, Huan Province (oleic acid, 52.05; linoleic acid, 20.09; linolenic acid, 2.3) [39]; Bama Mountain, Gaungxi (oleic acid, 31.76; linoleic acid, 47.42; linolenic acid, 0.25) [39]; Gaozhou, Guangdong (oleic acid, 78.4; linoleic acid, 6.97; linolenic acid, 0.41) [34]; Tengchong, Yunnan (oleic acid, 73.7; linoleic acid, 13.6; linolenic acid, 0.3) [40]; Jinhua, Zhejiang (oleic acid, 78.84; linoleic acid, 9.41; linolenic acid, 0.21) [41]; Zhangshu, Jiangxi (oleic acid, 79.45; linoleic acid, 8.59; linolenic acid, 0.29) [41] and Guangshan, Henan (oleic acid, 28.64; linoleic acid, 3.65; linolenic acid, 0.1) [42] (with all the above-listed regions having average altitudes far lower than the Weining region). *Camellia weiningensis* has a higher oleic acid content than *C*. *oleifera* in 80% of these regions, a lower linoleic acid content than *C*. *oleifera* in 75% of these regions and the second highest linolenic acid (following that of *C*. *oleifera* in Liuyang, Hunan). The comparative results did not show the particular advantage of *C*. *weiningensis* due to its high cultivation altitude. The content of saturated fatty acids in tea oil tends to increase with increasing latitude of the planting area, whereas that of unsaturated fatty acids exhibits the opposite trend [43, 44]. Between the altitudes of 297 m and 880 m, the oil content and chemical components of tea oil of wild *C*. *oleifera* vary greatly; however, the variations do not have noticeable associations with altitude [45]. Between altitudes of 1,900 and 2,500 m, the unsaturated fatty acid content of walnut oil shows a wave-like fluctuations but altitude has no noticeable influence on the oil’s linolenic acid content [46]. In green coffee beans, the four principal fatty acids, i.e., palmitic acid, linolenic acid, stearic acid and oleic acid, show moderately negative correlations with altitude [47]. Therefore, although the accumulation of oleic, linoleic and linolenic acids during seed development of *C*. *oleifera* varies greatly among altitudes, the differences are mainly attributed to differences in climatic factors, and the fatty acid composition of seeds is not significantly correlated with altitude. Thus, altitude cannot be used as an evaluation criterion for the fatty acid components of *C*. *oleifera* seeds.

The mechanisms of action of site conditions on the oil yield and fatty acid composition of *C*. *oleifera* have been reported. Soil organic matter (SOM) may serve as a limiting factor for oil yield, whereas available phosphorus (AP) deficiency is the main limiting factor for oil yield in the central subtropical regions of China [38]. During seed development, the relative activity of fatty acid desaturase 2 (FAD2), which is associated with the syntheses of oleic acid and linoleic acid, continuously decreases, whereas that of FAD3 gradually increases. The differences in the relative activity of FAD2 among regions of different altitude might be associated with variations in the average daily temperature and average daily temperature difference; suitable temperature, a large temperature difference and low precipitation all promote oil accumulation in seeds, thereby increasing the oil content [48, 49]. According to the literature, the net photosynthetic rate has a very significant correlation with oil content (P<0.001) and a significant correlation with the kernel rate (P<0.01). Acid rain significantly enhances the leaf photosynthetic rate of woody tree species [50]. In Weining, acid rain occurs frequently, which might constitute one cause of the higher oil yield of *C*. *weiningensis* than of *C*. *oleifera* in half the regions mentioned above. However, in this study, we did not explore the mechanism underlying the high oil yield of *C*. *weiningensis*, which may be an important direction for future research. Analysis of the mechanism of action each factor will undoubtedly benefit the improvement of site conditions for *C*. *weiningensis*, thereby enhancing the yield and quality of the tea oil.

In tea seed oil, direct transformation processes occur from palmitic acid to stearic acid, from stearic acid to oleic acid, from oleic acid to linoleic acid and from linoleic acid to linolenic acid, and the selection of a suitable fruit harvesting time can improve the proportion of unsaturated fatty acids in tea seed oil [48]). Therefore, the selection of the optimal fruit cropping time can bring the oil, oleic acid, linoleic acid, and linolenic acid contents and fresh seed and kernel rates under balanced control [51]. Late August to early September is a suitable harvest time for *C*. *oleifera* in Honghua, Zhejiang [52]. With the maturation of *C*. *oleifera* seeds, the unsaturated fatty acid content in seed oil increases and the levels of antioxidants, such as vitamin E and β-sitosterol, increase; therefore, the time after October and before fruit fall is the best fruit-harvesting time for *C*. *oleifera* [53].

In this study, we comprehensively assessed 45 superior *C*. *weiningensis* cultivars using PCA and gray correlation analysis and screened nine superior cultivars for breeding. In addition, the coefficients of genetic variation for oleic acid, transverse diameter and arachidonic acid were all lower than 10%, which has not been reported before. Presumably, the low levels of variation in these components are due to SOM content, harvest time, high altitude and frequent acid rain. However, to verify this presumption, additional systematic research needs to be conducted in the future. In addition, currently, the popularization of *C*. *weiningensis* is at the superior variety selection stage, and mass production has not yet begun. Therefore, other indices to evaluate the superiority of the selected varieties, such as unit output, cannot yet be calculated; this topic constitutes another important direction for further research.
